# Cationic Site-Preference in the Yb_14-*x*_Ca*_x_*AlSb_11_ (4.81 ≤ *x* ≤ 10.57) Series: Theoretical and Experimental Studies

**DOI:** 10.3390/ma9070553

**Published:** 2016-07-08

**Authors:** Gnu Nam, Eunyoung Jang, Hongil Jo, Mi-Kyung Han, Sung-Jin Kim, Kang Min Ok, Tae-Soo You

**Affiliations:** 1 Department of Chemistry, Chungbuk National University, Cheongju, Chungbuk 28644, Korea; ggechu1@hanmail.net (G.N.); s2jeyoung@hanmail.net (E.J.); 2 Department of Chemistry, Chung-Ang University, Seoul 06974, Korea; hongil55@hanmail.net (H.J.); kmok@cau.ac.kr (K.M.O.); 3 Department of Chemistry and Nano Science, Ewha Woman University, Seoul 03760, Korea; mikihan@ewha.ac.kr (M.-K.H.); sjkim@ewha.ac.kr (S.-J.K.)

**Keywords:** Zintl phases, single-crystal X-ray diffraction, site preference, electronic structure, mixed cations

## Abstract

Four quaternary Zintl phases with mixed-cations in the Yb_14-x_Ca_x_AlSb_11_ (4.81 ≤ x ≤ 10.57) series have been synthesized by using the arc-melting and the Sn metal-flux reaction methods, and the isotypic crystal structures of the title compounds have been characterized by both powder and single-crystal X-ray diffraction (PXRD and SXRD) analyses. The overall crystal structure adopting the Ca_14_AlSb_11_-type can be described as a pack of four different types of the spiral-shaped one-dimensional octahedra chains with various turning radii, each of which is formed by the distorted ((Yb/Ca)Sb_6_) octahedra. Four symmetrically-independent cationic sites contain mixed occupations of Yb^2+^ and Ca^2+^ with different mixing ratios and display a particular site preference by two cationic elements. Two hypothetical structural models of Yb_4_Ca_10_AlSb_11_ with different cationic arrangements were designed and exploited to study the details of site and bond energies. QVAL values provided the rationale for the observed site preference based on the electronegativity of each atom. Density of states (DOS) curves indicated a semiconducting property of the title compounds, and crystal orbital Hamilton population (COHP) plots explained individual chemical bonding between components. Thermal conductivity measurement was performed for Yb_8.42(4)_Ca_5.58_AlSb_11_, and the result was compared to compounds without mixed cations.

## 1. Introduction

One of the smart ways to lower global energy consumption is to recover wasted heat produced by industries or automobiles and to convert it into electricity. Thermoelectric (TE) materials are well suited for this purpose and can be exploited in various energy recovery, as well as conversion systems using the Seebeck effect [1,2,3,4,5]. Besides research activities to improve the efficiency of conventional thermoelectric materials [6,7,8,9], there have been also various studies to seek for novel thermoelectric material candidates. The Zintl phase is one of the promising candidates to fulfill this demand due to its intrinsically suitable properties, such as complex crystal structures and semiconducting properties. Among several Zintl phase compounds considered as potential thermoelectric materials, Yb_14_MnSb_11_ is one of the best *p*-type TE material for the high temperature applications [10]. In order to improve its figure of merit *zT* value even further, various cationic or anionic element substitutions have been attempted, respectively, to lower the lattice thermal conductivity or to optimize the carrier concentration [11,12,13,14,15]. 

During our systematic investigations to look for novel Zintl phase thermoelectric materials [16,17,18,19], we have successfully synthesized four quaternary compounds belonging to the Yb_14-*x*_Ca*_x_*AlSb_11_ system. Initially, we expected to lower the lattice thermal conductivity of the system by introducing the Ca substitution for Yb. However, we interestingly found that two cationic elements of Yb^2+^ and Ca^2+^ having very similar cationic sizes displayed a particular site preference over four available cationic sites. In the case like ours, where the size of the substituting element was similar to the original element, the observed site preference should be elucidated by the electronic factor.

In this article, we report four isotropic quaternary compounds belonging to the Yb_14-*x*_Ca*_x_*AlSb_11_ (4.81(3) ≤ *x* ≤ 10.57(2)) series and adopting the Ca_14_AlSb_11_-type structure [20]. The overall crystal structure was alternatively illustrated as a pack of four different types of cationic coordination environments forming the spiral-shaped 1D octahedral chain. A series of theoretical calculations was performed by using the tight-binding linear muffin-tin orbital (TB-LMTO) method to understand the experimentally-observed cationic site preference, as well as the overall electronic structure. Two structural models with different atomic arrangements were designed for the calculations, and the results were thoroughly analyzed via the electronic factor criterion based on the QVAL values. Density of states (DOS) and crystal orbital Hamilton population (COHP) curves were also studied. The temperature-dependent thermal conductivity was measured, and SEM images of nicely grown single crystals are provided, as well, in this article.

## 2. Results and Discussion

### 2.1. Crystal Structure Analysis

**Figure 1:**
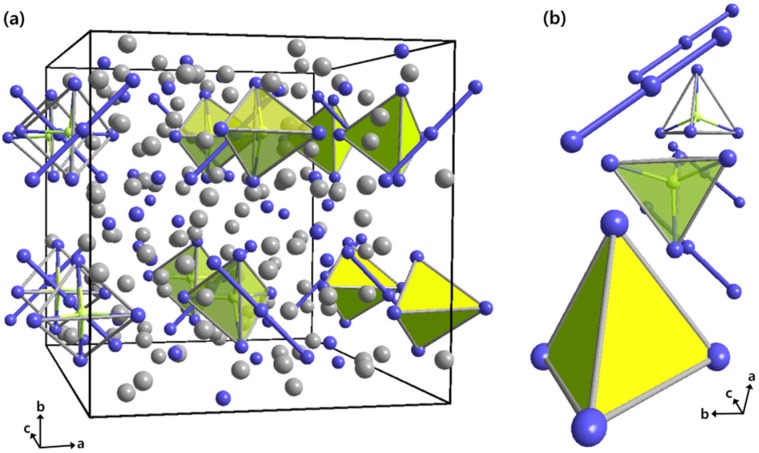
(**a**) Combined ball-and-stick and polyhedral representations of the crystal structure of the Yb_14-*x*_Ca*_x_*AlSb (4.81(3) ≤ *x* ≤ 10.57(2)) series view along the *c*-axis direction; (**b**) The (AlSb_4_)^9−^ tetrahedra and (Sb_3_)^7−^ linear trimers are highlighted in green and blue, respectively. A unit cell is outlined in black, and the color codes are as follows: *M*, gray; Al, green; Sb, blue.

Four quaternary compounds in the Yb_14-*x*_Ca*_x_*AlSb_11_ (4.81(3) ≤ *x* ≤ 10.57(2)) series have been synthesized by using the Sn metal-flux method, and four isotypic crystal structures were characterized by both powder and single-crystal X-ray diffraction (PXRD and SXRD) experiments. The title compounds crystallized in the Ca_14_AlSb_11_-type structure [20] having a tetragonal space group *I*4_1_/*acd* (Pearson code *tI*208, *Z* = 8), as shown in Table 1. There existed nine crystallographically-independent atomic sites, including four Yb/Ca mixed sites, one Al site and four Sb sites in a unit cell in which a total of 208 atoms were located (Table S1). The overall crystal structure of the Ca_14_AlSb_11_-type phase has been previously described by many researchers as a complex mixture of two major anionic building moieties: one (AlSb_4_)^9−^ tetrahedron and one (Sb_3_)^7−^ linear trimer, which were alternately packed together along the *c*-axis direction, with four isolated Sb^3−^ anionic sites and 14 isolated Ca sites in each formula unit. Therefore, the crystal structure of our four isotypic compounds can simply be described by following the same view point as the previous reports, except 14 cationic positions now occupied by Yb^2+^, as well as Ca^2+^ (Figure 1).

**Figure 2:**
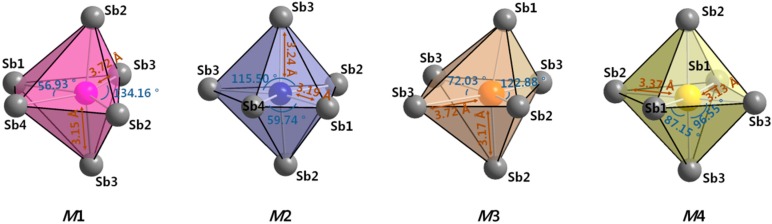
Four octahedral cation sites in the Yb_14-*x*_Ca*_x_*AlSb (4.81(3) ≤ *x* ≤ 10.57(2)) series displayed as coordination polyhedra with surrounding six Sb atoms. Selected lengths and angles are also displayed.

However, if we take a look at the given crystal structure from the perspective of the cationic polyhedron, the crystal structure of the title phase can alternatively be viewed as a pack of four different types of distorted ((Yb/Ca)Sb_6_) octahedra. The geometrical features of these four octahedra closely resemble the structural characteristics of those in the previously-reported Yb_14-*x*_Ca*_x_*MnSb_11_ series [14], as illustrated in Figure 2. For instance, the *M*1-site provides the significantly distorted octahedral coordination environment, which can accommodate the relatively larger-sized element, whereas the *M*4-site serves as the most symmetric coordination environment among the four cationic sites, which is suitable only for the relatively smaller-sized atom.

**Figure 3:**
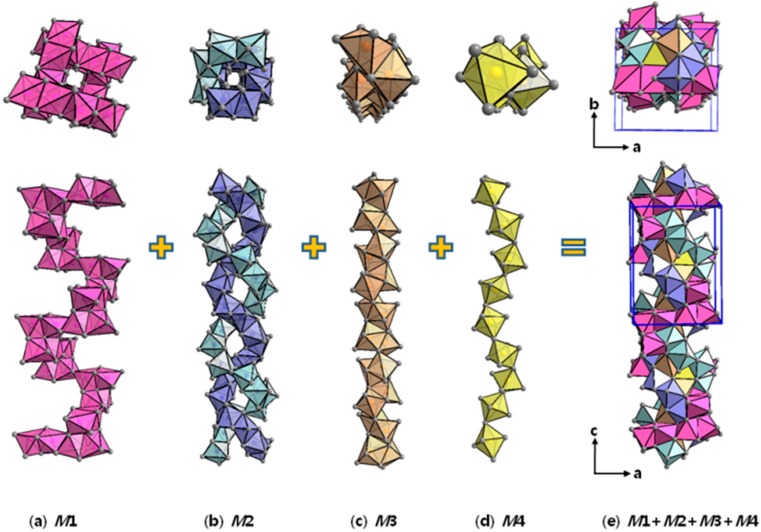
Schematic illustration showing the formation of the Yb_14-*x*_Ca*_x_*AlSb (4.81(3) ≤ *x* ≤ 10.57(2)) series (Ca_14_AlSb_11_-type) described as a combination of four different types of spiral-shaped 1D octahedra chains. (**a**) *M*1; (**b**) *M*2; (**c**) *M*3; (**d**) *M*4; (**e**) *M*1 + *M*2 + *M*3 + *M*4.

More interestingly, as shown in Figure 3, the same type of octahedra can be connected to each other along the crystallographic *c*-axis direction via corner-, edge- or face-sharing and eventually form four different types of the one-dimensional (1D) spiral-shaped octahedra chains. The *M*1-chain shows the largest turning radius; two of the *M*2-chains are twisted with each other with intermediate turning radii; and the *M*3- and *M*4-chains display the second smallest and the smallest turning radius, respectively (Figure 3a–d). All of these 1D spiral-chains eventually get coiled with each other, resulting in one thick rope-like chain (Figure 3e).

The substituting Ca^2+^ cations are found all over the four Yb^2+^ sites with different partial occupations varying from ca. 31%–78%, as listed in Table S1. In addition, the bond distances between Al and Sb1 in the (AlSb_4_)^9−^ tetrahedron and between Sb3 and Sb4 in the (Sb_3_)^7−^ are linear trimer ranged, respectively, from 2.712–2.714 Å and from 3.190–3.193 Å (Table S2), which are comparable to 2.718 Å and 3.196 Å found in Ca_14_AlSb_11_ [20] and 2.749–2.762 Å and 3.169–3.183 Å in the Yb_14-*x*_Ca*_x_*MnSb_11_ (2 ≤ *x* ≤ 8) series [14], respectively. Moreover, despite the nearly identical ionic size of Yb^2+^ and Ca^2+^ cations (1.13 Å vs. 1.06 Å) [21], the increase of Ca content in the formula from 4.81–10.57 resulted in the gradual increase of lattice parameters *a* and *c* from 16.5704 to 16.6488 Å and from 22.1976 to 22.3684 Å, respectively (Table 1). This kind of unit cell expansion caused by the Ca^2+^ substitution for Yb^2+^ has been also reported in the Yb_14-*x*_Ca*_x_*MnSb_11_ (0 ≤ *x* ≤ 14) series [14], as well as several other Zintl phases, including YbZn_2_Sb_2_ [22] and Yb_11_GaSb_9_ [23]. Prof. Kauzlarich provided the rationale for this observation based on the more ionic bond character of Ca-Sb than Yb-Sb, which was descended from the electronegativity (EN) difference between Yb and Ca (EN(Yb) = 1.06 vs. EN(Ca) = 1.04, Allred–Rochow scale) [24] in the article about the Yb_14-*x*_Ca*_x_*MnSb_11_ (0 ≤ *x* ≤ 14) series [14]. In addition, the relatively lighter Ca^2+^ substitution for the heavier Yb^2+^ (40.08 g/mol vs. 173.04 g/mol) in our Yb_14-*x*_Ca*_x_*AlSb_11_ (4.81 ≤ *x* ≤ 10.57) series dramatically lowered the densities of compounds from 6.864 down to 5.107 g/cm^3^, respectively, for *x* = 4.81 and *x* = 10.57. This type of cation substitution can introduce a large atomic mass disordering to the series, and it may eventually result in a reduction of thermal conductivity. The measurement result of thermal conductivity will be provided later in this article.

The chemical formula of the title compounds can be charge-balanced by adopting the Zintl–Klemm concept as follows, ((Yb_14-*x*_Ca*_x_*)^2+^)_14_((AlSb_4_)^9−^(Sb_3_)^7−^(Sb^3−^)_4_), similar to the previous reports [10,11,12], where 14 Yb^2+^/Ca^2+^ cations are considered as divalent by donating a total of 28 electrons to the anionic framework composed of the (AlSb_4_)^9−^ tetrahedron, the (Sb_3_)^7−^ linear trimer and the isolated (Sb^3−^)_4_. The suggested charge-balanced chemical formula is confirmed by a band-gap at the Fermi level (*E*_F_) in DOS curve analysis. The further discussion about the overall electronic structure will be discussed in the consecutive section.

### 2.2. Site Preference and Electronic Structure

To understand further details about the overall electronic structure of the title compounds and the site preference between two cationic elements over four available cationic sites, a series of theoretical calculations by using the TB-LMTO method with the atomic sphere approximation (ASA) has been carried out [25,26,27,28,29]. As briefly mentioned in the Crystal Structure Analysis section, two cationic elements of Yb and Ca displayed a particular site preference over four available cationic sites in the isotypic crystal structures: the *M*2 (*M*3) site contained the largest Yb (Ca) content. 

Firstly, in order to find out the energetic influence of the location of Yb and Ca in the crystal structure for the total electronic energy, we have designed two hypothetical model structures having particular Yb and Ca arrangements at four available cationic sites. Therefore, Model 1 contained the Yb atom only at the *M*2 site, where the largest Yb (or smallest Ca) content was experimentally refined, whereas Model 2 contained the Yb atom only at the *M*3 site, where the smallest Yb (or largest Ca) content was observed. In both models, the rest of the three cationic sites were occupied only by Ca atoms, resulting in an idealized composition of Yb_4_Ca_10_AlSb_11_. A series of theoretical calculations were conducted by using two hypothetical models, and the resultant total electronic energy of each model was compared. As shown in Table 2, the total energy of Model 1 was smaller than that of Model 2 by 8.15 eV/f.u., which was in a good agreement with our experimental observation. To understand the origin of this energy difference, the detailed site and the bond energies of both models were further analyzed. Firstly, the bond energies of six selected interatomic interactions in two models, including *M*1-Sb and *M*2-Sb, showed a small total energy difference of 0.11 eV/f.u. On the other hand, the site energies of nine different atomic sites for two models displayed a relatively larger energy difference of ca. 6 eV/f.u. In particular, the Yb occupation either at the *M*2 or the *M*3 site, respectively, in Model 1 or Model 2 raised the site energy of the site, which was energetically unfavorable for both models. However, the site energies of four Sb atoms surrounding the *M*2 or the *M*3 sites were relatively smaller in Model 1 than those in Model 2. Therefore, the site energy differences between two different models should be considered as a major contribution to a total electronic energy difference between Model 1 and Model 2.

Secondly, this type of cationic site preference caused by the site energy differences can be explained either by the size factor based on the substituting atomic sizes or the electronic factor based on the quantity called the “QVAL value” [30]. The size-factor was originally applied to the Eu_13_*A*MnSb_11_ series (*A* = Ca, Sr, Ba, Yb) [31] to rationalize the observed site-preference between Eu and *A*. In the article, two types of contrary site preferences caused by four different substituting cations, whether their cationic sizes were relatively smaller (Ca and Yb) or larger (Sr and Ba) than Eu, were nicely interpreted by the degree of geometrical distortions of four different ((Eu/*A*)Sb_6_) octahedra. For instance, the relatively larger Sr^2+^ and Ba^2+^ cations preferred to occupy the most distorted octahedral sites (*M*4 site), whereas the relatively smaller Ca^2+^ and Yb^2+^ cations preferred to occupy the less distorted (in other words, more symmetric) cationic sites, such as the *M*3 site. Such a criterion based on the size factor was also applied to the site preference between two cations with nearly the same sizes in the Yb_14-*x*_Ca*_x_*MnSb_11_ (*x* = 2~8) series [14]. The rationale for this observation was attributed to the fact that the stronger ionic character of Ca^2+^ made it keep a longer bond distance to the surrounding Sb atom than Yb^2+^ did despite the nearly same cationic radii of those (*r*(Ca^2+^) = 1.06 Ǻ; *r*(Yb^2+^) = 1.13 Ǻ) [21].

In the situations where two similarly-sized elements occupy the same atomic sites, the electronic factor based on the so-called “QVAL value” [30] can also provide a rationale for such a complex site preference between components. The QVAL value can be evaluated for a particular atomic site in a unit cell by evaluating the summation of integrated electron densities inside the corresponding Wigner–Seitz (WS) sphere. As a result, we can eventually expect an element with a larger electronegativity to occupy the atomic site having a larger QVAL value. In the past several years, numerous observed site preferences between components in Zintl phases and polar intermetallic compounds have been nicely explained by this QVAL value criterion. Some examples include the EuZn*_x_*In_4-*x*_ (*x* = 1.1~1.2) [32], the *RE*_4_LiGe_4_ (*RE* = La, Ce, Pr and Sm) [33], the *RE*_11_Ge_4_In_6-*x*_M*_x_* (*RE* = La, Ce; M = Li, Ge; *x* = 1, 1.96) series [17] and BaLi_1.09(1)_In_0.91_Ge_2_ [34].

To find out the QVAL value for each atomic site in the title compounds, we initially exploited Ca_14_AlSb_11_ [20] for the TB-LMTO calculation, and the resultant QVAL values were evaluated as displayed in Table 3. Among four cationic sites, the largest QVAL value was observed at the Ca2 site, whereas the smallest one was found at the Ca3 site. The values for the Ca1 and the Ca4 sites were similar to each other and intermediate between those of the Ca2 and the Ca3 sites. These results implied that the more electronegative element would prefer to occupy the Ca2 site first, whereas the Ca3 site would be the least favorable site among the four available cationic sites. Our experimental results were in very good agreement with this criterion that the slightly more electronegative Yb atom preferred to occupy the Ca2 site first, the Ca1 and Ca4 sites seconds and the Ca3 site last.

In addition, this type of site preference analysis based on the electronic factor can also be applied to the previously reported Yb_14-*x*_Ca*_x_*MnSb_11_ series [14], where Yb^2+^ and Ca^2+^ cations displayed similar occupation factors to the present work over four cationic sites, and very nicely explains the experimentally-refined site preference of two cation elements. Therefore, this theoretical approach again confirms that if there is a large size difference between elements, the size factor works as a dominant factor to determine the site preference. On the other hand, if the size difference is subtle, the electronic factor based on the QVAL value criterion should be a decisive factor for the site preference, as proven in this work, as well as many other previous reports [30,35,36].

**Figure 4:**
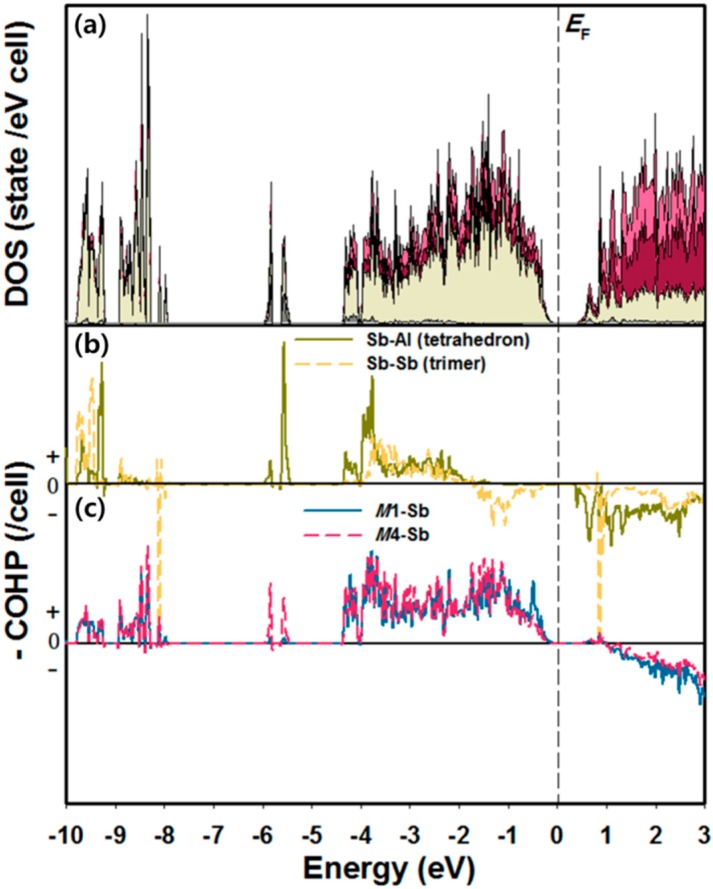
Density of states (DOS) and crystal orbital Hamilton population (COHP) curves for Yb_8_Ca_6_AlSb_11_. (**a**) Total DOS (the most outline), Yb (red region), Ca (pink region), Al (gray region) and Sb (ivory region). COHP curves represent; (**b**) the averaged Sb-Al interactions in the (AlSb_4_)^9−^ and the averaged Sb-Sb interactions in the (Sb_3_)^7−^; as well as (**c**) the *M*1-Sb and the *M*4-Sb interactions of two different (*M*Sb_6_) octahedra environments. *E*_F_ (dashed vertical line) is the energy reference (0 eV).

Lastly, DOS and COHP analyses have been performed based on the calculation results using Yb_8_Ca_6_AlSb_11_. As can be seen in Figure 4a, the total DOS (TDOS) curve displays an overall orbital mixing of four components, particularly between ca. −4.5 eV and *E*_F_. The band-gap observed at *E*_F_ implies a semiconducting property of the title compound. In particular, in the valence band region of DOS curves, the region between −10 and −8 eV contains a major contribution from Sb 5s state with a small contribution from Al 3s state, and the region between −4.5 eV and *E*_F_ includes a mixed contributions from Sb 5p and Al 3p states with some portions from Yb and Ca states. The conduction band region above *E*_F_ is mainly dominated by Yb 5d and Ca 3d states. The averaged Sb-Al COHP curve representing the interatomic interaction of the (AlSb_4_)^9−^ tetrahedron is optimized at *E*_F_, whereas the averaged Sb-Sb COHP curves for the (Sb_3_)^7−^ linear trimer show some portions of an anti-bonding character at *E*_F_ (Figure 4b). However, this unfavorable interatomic interaction is overridden by several strong bonding characteristics of the averaged *M*-Sb COHP curves for the octahedral coordination environments (Figure 4c).

### 2.3. Thermal Transport Property

Due to a complex crystal structure with as many as 208 atoms in the unit cell and a large mass disordering between two cations caused by the Ca substitution for Yb, a reduced thermal conductivity was expected for the title compounds.

**Figure 5:**
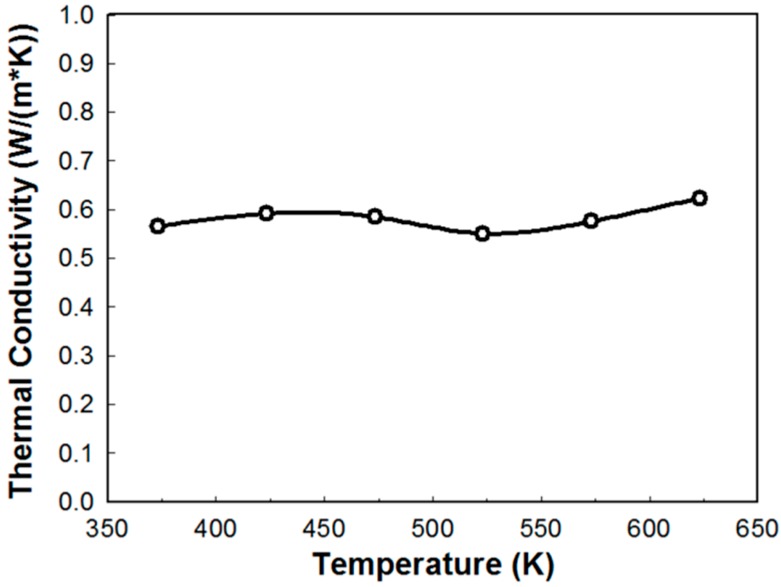
Temperature dependence of the thermal conductivity of Yb_8.41(4)_Ca_5.58_AlSb_11_.

The total thermal conductivity of Yb_8.21(3)_Ca_5.79_AlSb_11_ was measured in the temperature range between 373 K and 623 K, and the resultant values were plotted in Figure 5. The nearly flat values of thermal conductivity varying between 0.55 and 0.62 W/mK were observed in the temperature range, and these values were slightly smaller than those of several already reported Ca_14_AlSb_11_-type compounds, such as the Yb_14_Mn_1-*x*_Al*_x_*Sb_11_ (0.5~1.0 W/mK) [11], the Yb_14-*x*_Ca*_x_*MnSb_11_ (0.6~1.1 W/mK) [14] and the Yb_14_Mn_1-*x*_Zn*_x_*Sb_11_ (0.7~1.1 W/mK) series [12]. Unlike the other many Zintl phases, which usually displayed a reduced thermal conductivity as temperature increased due to the phonon scattering by thermal vibrations, a slight increasing of thermal conductivity at the high temperature might be attributed to the onset of bipolar conduction between the electron and hole. However, the electrical resistivity was not measured for this sample; it could not be definitely concluded whether the relatively low thermal conductivity was originated from the electronic contribution or phonon scattering effect by the lattice.

## 3. Materials and Methods

### 3.1. Synthesis

**Figure 6:**
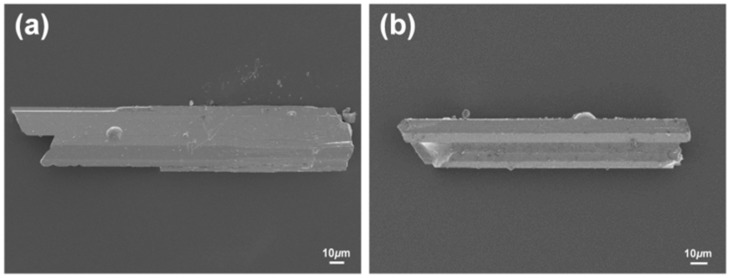
SEM images of needle-shaped single crystals of (**a**) Yb_9.19(3)_Ca_4.81_AlSb_11_ and (**b**) Yb_8.42(2)_Ca_5.58_AlSb_11_.

All sample preparation processes were performed in an Ar-filled glove-box with O_2_ and H_2_O contents below 0.1 ppm or under vacuum. Reactant elements were used as purchased from Alfa Aesar (Ward Hill, MA, USA) (Yb, ingot, 99.9 wt %; Ca, shot, 99.5 wt %; Al, piece, 99.9 wt %; and Sn, tear drop, 99.8 wt %). The slightly tanned surfaces of Yb and Ca were cleaned by scrapping off using a scalpel or a metal brush before being loaded into a reaction container. The title Yb_14-*x*_Ca*_x_*AlSb_11_ phase was originally obtained from the arc-melting reaction using the molar ratio of Yb:Ca:Al:Sb = 4:2:2:6 to target the Yb_3-*x*_Ca*_x_*AlSb_3_ phase. However, once the title phase was verified, we attempted to produce the single-phase product of the Yb_14-*x*_Ca*_x_*AlSb_11_ phase by using the Sn-metal flux reaction with the molar ratio of Yb:Ca:Al:Sb:Sn = 14−*x*:*x*:6:11:86 (*x* = 4~10). Each mixture of four elements with various elemental ratios was loaded in a 2 cm^3^ alumina crucible, and the elemental Sn metals were placed on top and at the bottom of the mixture of other elements. After that, each reaction container was subsequently enclosed in an evacuated fused-silica jacket to protect it from the oxidation during the high temperature reaction. All reactions followed the temperature profile shown below: the reaction ampoules were initially heated to 500 °C in 2 h using a box-furnace, kept there for 2 h. After then, those were heated up to 1000 °C in 2 h, held there for 6 h and then cooled down to 700 °C at the rate of 3 °C/h. At last, the reaction ampoules were taken out of a furnace at 700 °C and instantaneously centrifuged to separate the remaining molten Sn metals from the single crystals. The nicely grown needle-shaped single crystals displayed shiny metallic luster, as observed in scanning electron microscope (SEM) images (Figure 6), and were stable under the ambient conditions for several days.

### 3.2. Powder and Single-Crystal X-ray Diffraction Experiments

Four title compounds have been characterized by both powder and single-crystal X-ray diffractions. PXRD patterns were obtained using a Bruker D8 diffractometer (Billerica, MA, USA) equipped with an area detector and monochromatic Cu K*α*_1_ radiation (*λ* = 1.54059 Å) at room temperature. The collected step size was set at 0.05° in the range of 15° ≤ 2*θ* ≤ 85° with a total exposure time of 1 h. Primarily, the phase purity of the title phase was checked by comparing the collected powder patterns with the simulated patterns, and then, all peaks in each pattern were indexed by using the program Rietica [37] to verify the lattice parameters of each unit cell. SXRD data were collected using the Bruker SMART APEX2 CCD-based diffractometer (Billerica, MA, USA) equipped with Mo K*α*_1_ radiation (*λ* = 0.71073 Å). Several silvery lustrous needle-shaped single-crystals were initially selected from each batch of crushed products and briefly checked for their qualities by a rapid scan. The best crystals were chosen for the full data collection by using Bruker’s APEX2 program [38]. Data reduction, integration and unit cell parameter refinements were conducted by using the SAINT program [39], and SADABS was used to perform semi-empirical absorption correction based on equivalents [40]. The entire sets of reflections of four title compounds were well matched with the tetragonal crystal system, and the space group *I*4_1_/*acd* was eventually selected for all crystal structures. Detail crystal structures were solved by direct methods and refined to convergence by full matrix least-squares methods on *F*^2^. Refined parameters include the scale factor, the atomic positions with anisotropic displacement parameters, extinction coefficients and occupancy factors for the Yb/Ca mixed site. During the last stage of a refinement cycle, atomic positions were standardized using STRUCTURE TIDY (Utrecht University, Utrecht, The Netherlands) [41]. Important crystallographic data, atomic positions with the atomic displacement parameters and selected interatomic distances are shown in Table 1 and Tables S1 and S2. Further details about each crystal structure can be obtained from the Fachinformationszentrum Karlsruhe, Eggenstein-Leopoldshafen, Germany (fax: (49) 7247-808-666; e-mail: crysdata@fiz-karlsruhe.de), depository number CSD-431298 for Yb_9.19(3)_Ca_4.81_AlSb_11_, CSD-431300 for Yb_8.42(4)_Ca_4.81_AlSb_11_, CSD-431299 for Yb_5.12(2)_Ca_8.98_AlSb_11_ and CSD-431301 for Yb_3.43(2)_Ca_10.57_AlSb_11_.

### 3.3. Electronic Structure Calculations

To understand the overall electronic structure and chemical bonding of the title compounds, the series of TB-LMTO-ASA calculations were conducted by using the Stuttgart TB-LMTO47 program (Max-Planck-Institut für Festkörperforschung, Stuttgart, Germany) [25,26,27,28,29]. For practical reasons, the idealized compositions of “Yb_4_Ca_10_AlSb_11_” and “Yb_8_Ca_6_AlSb_11_” were exploited, respectively, for two structural models to perform the site and the bond energy analyses and for one model to investigate the DOS and COHP curves. Exchange and correlation were treated by the local density approximation (LDA) [25,26,27,28,29]. Except spin-orbit coupling, all relativistic effects were taken into account using a scalar relativistic approximation. In the ASA method, space is filled with overlapping Wigner–Seitz (WS) atomic spheres (Max-Planck-Institut für Festkörperforschung, Stuttgart, Germany) [25,26,27,28,29]. The symmetry of the potential is considered spherical inside each WS sphere, and a combined correction is used to take into account the overlapping part [42]. The radii of WS spheres were calculated by requiring that the overlapping potential be the best possible approximation to the full potential and were determined by an automatic procedure [42]. This overlap should not be too large, because the error in kinetic energy introduced by the combined correction was proportional to the fourth power of the relative sphere overlap. No empty spheres were necessary.

The used WS radii for Yb_4_Ca_10_AlSb_11_ and Yb_8_Ca_6_AlSb_11_ are as follow: Yb = 1.91–1.96 Å, Ca = 1.90–1.96 Å, Al = 1.57 Å and Sb = 1.74–1.95 Å. The basis sets included 6s, 6p and 5d orbitals for Yb; 4s, 4p and 3d orbitals for Ca; 3s, 3p and 3d orbitals for Al; and 5s, 5p, 5d and 4f orbitals for Sb. The Yb 6p, Ca 4p, Al 3d and Sb 5d and 4f orbitals were treated by the Löwdin downfolding technique [43]. The *k*-space integrations were conducted by the tetrahedron method [44], and the self-consistent charge density was obtained using 163 and 262 irreducible *k*-points in the Brillouin zone of Yb_4_Ca_10_AlSb_11_ and Yb_8_Ca_6_AlSb_11_, respectively.

### 3.4. Thermal Conductivity Analysis

The thermal conductivity of Yb_8.21(3)_Ca_5.79_AlSb_11_ was measured by using the thermal diffusivity, *κ* = *D* × *C_p_* × *ρ*, where *ρ*, *D* and *C_p_* are the density, thermal diffusivity and specific heat, respectively. The polycrystalline pellet sample was prepared by spark plasma sintering with the compactness >95%. The thermal diffusivity and specific heat were measured by the flash diffusivity-heat capacity method using a NETZSCH LFA 457 MicroFlash™ instruments (NETZSCH, Selb, Germany). In the flash method, the front face of a disc-shaped plane-parallel surface was heated by a laser beam. The thermal response on the rear surface was monitored using an infrared detector, and the thermal diffusivity was calculated from the time delay and the shape of the temperature rise.

## 4. Conclusions

Four quaternary Zintl phases in the Yb_14-*x*_Ca*_x_*AlSb_11_ (4.81 ≤ *x* ≤ 10.57) series have been successfully synthesized by using both arc-melting and Sn metal-flux methods. Two cationic elements of Yb^2+^ and Ca^2+^ showing mixed occupations proved that the title phase could accommodate the wide range of mixing ratios between two cations. The isotypic crystal structures were visualized through a perspective based on four different cationic coordination environments and interpreted as a pack of four types of spiral-shaped 1D octahedra chains with various turning radii. Each 1D octahedral chain was composed of the corner-, edge-, or face-sharing distorted [(Yb/Ca)Sb_6_] octahedra. TB-LMTO calculations using two hypothetical structural models of Yb_4_Ca_10_AlSb_11_ with different cationic arrangements proved that the overall stability of a crystal structure should be attributed to the site energies of four Sb sites surrounding the *M*2 sites. QVAL values for each atomic site in a model structure proved that the more electronegative Yb atom should prefer the *M*2 site with the largest QVAL value, whereas the more electropositive Ca atom should prefer the *M*3 site. In addition, our theoretical calculations proved that the electronic factor based on the QVAL value criterion can be the critical factor for the site preference when there was a small size difference between the substituting and the host elements. The overall DOS curves indicated a semiconducting property of the title phase with a small band-gap, and the Sb-Al and *M*-Sb COHP curves explained the overall stability of bonding interactions in the given structural type. The Ca substitution for Yb was insignificant to lower the overall thermal conductivity in Yb_8.42(2)_Ca_5.58_AlSb_11_, but the measured value was still comparable to those of previously reported compounds in the Yb_14-*x*_Ca*_x_MT*_11_ (*M* = Al, Mn; *T* = Sb, Bi) system.

## Figures and Tables

**Table 1:** Single crystal X-ray diffraction data and structure refinement results for the Yb_14-*x*_Ca*_x_*AlSb_11_ (4.81(3) ≤ *x* ≤ 10.57(2)) series.

Chemical Compositions		Yb_9.19(3)_Ca_4.81_AlSb_11_	Yb_8.42(4)_Ca_5.58_AlSb_11_	Yb_5.12(2)_Ca_8.98_AlSb_11_	Yb_3.43(2)_Ca_10.57_AlSb_11_
Formula weight (g/mol)		3149.25	3046.87	2640.45	2383.40
space group; *Z*		*I*4_1_/*acd*; 8			
Lattice parameters Å	*A*	16.5704(5)	16.6366(8)	16.6374(6)	16.6488(2)
	*C*	22.1967(7)	22.2244(12)	22.3365(8)	22.3684(3)
Volume (Å^3^)		6094.7(4)	6151.2(7)	6182.8(5)	6200.1(2)
*d*_calcd_ (g/cm^3^)		6.864	6.580	5.609	5.107
*θ* range for data collection		2.90°–27.86°	2.89°–26.37°	2.45°–29.13°	2.45°–28.27°
Reflections collected		73,570	28,005	22,936	22,847
Data/restraints/parameters		1826/0/65	1579/0/65	2086/0/65	1929/0/65
GOF on *F*^2^		1.075	1.044	1.177	1.227
*R* ^1^ indices (all data)	*R*_1_	0.0364	0.0498	0.0319	0.0307
	*wR*_2_	0.0427	0.0590	0.0565	0.0600
Largest differences of peak/hole (e/Å^3^)		1.196/−1.283	1.356/−1.335	1.183/−2.946	1.020/−3.155

**^1^** *R*_1_ = Σ||*F*_o_| − |*F*_c_||/Σ|*F*_o_|; *wR*_2_ = (Σ(*w*(*F*_o_^2^ − *F*_c_^2^)/Σ(*w*(*F*_o_^2^)^2^))^1/2^, where *w* = 1/(*σ*^2^*F*_o_^2^ + (A-*P*)^2^ + B-*P*), and *P* = (*F*_o_^2^ + 2*F*_c_^2^)/3; *A* and *B*: weight coefficients.

**Table 2:** Results of tight binding analysis of total energy and band energy (eV) in Model 1 and Model 2 for Yb_4_Ca_10_AlSb_11_.

Hypothetical Structures	Model 1	Model 2
*E*_Total_	0	8.15
*E*_Band_	0	6.57
**Site energies**		
Ca1	105.41	106.88
*M*2 ^a^	162.28	137.92
*M*3 ^b^	101.00	124.25
Ca3	56.00	56.00
Al	−52.21	−52.32
Sb1	−1089.05	−1086.61
Sb2	−1014.10	−1013.22
Sb3	−517.63	−517.46
Sb4	−244.61	−242.43
Total	−2492.90	−2486.98
**Bond energies**		
Ca1-Sb	−4.62	−4.64
*M*2 ^a^-Sb	−4.81	−4.59
*M*3 ^b^-Sb	−4.51	−4.77
Ca3-Sb	−5.08	−5.09
Al-Sb1 (tetrahedron)	−11.27	−11.29
Sb3-Sb4 (trimer)	−1.85	−1.88
Total	−32.16	−32.27
Site energies + bond energies	−2525.06	−2519.25
Relative total	0	6.81

*^a^* *M*2 = Yb for Model 1, Ca2 for Model 2; *^b^ M*3 = Ca2 for Model 1, Yb for Model 2.

**Table 3:** QVAL values for each atomic site of Ca_14_AlSb_11_.

Atom	Ca1	Ca2	Ca3	Ca4	Al	Sb1	Sb2	Sb3	Sb4
Wyckoff site	32*g*	32*g*	32*g*	16*e*	8*a*	32*g*	32*g*	16*f*	8*b*
QVAL	1.746	1.821	1.659	1.759	2.843	5.323	4.597	5.400	5.322

## Supplementary Materials

The supplementary materials are available online at www.mdpi.com/1996-1944/9/7/553/s1.
