# Supplementary material for: Cationic Site-Preference in the Yb14-xCaxAlSb11 (4.81 ≤ x ≤ 10.57) Series: Theoretical and Experimental Studies
Source: Materials (Basel). 2016 Jul 8;9(7):553. doi: 10.3390/ma9070553 (PMC5456874; doi:10.3390/ma9070553)
Supplement: Supplementary file 1 [file materials-09-00553-s001.pdf]

Supplementary Materials: Cationic Site-Preference in the $Yb_{14-x}Ca_xAlSb_{11}$ (4.81 ≤ x ≤ 10.57) Series: Theoretical and Experimental Studies

Gnu Nam, Eunyoung Jang, Hongil Jo, Mi-Kyung Han, Sung-Jin Kim, Kang Min Ok and Tae-Soo You

Table S1. Atomic coordinates and equivalent isotropic displacement parameters (U_{eq} [a]) from single-crystal structure refinements for the $Yb_{14-x}Ca_xAlSb_{11}$ (4.81(3) ≤ x ≤ 10.57(2)) series.

Atom	Wyckoff Site	Occupation	*x*	*y*	*z*	U_{eq} [a] (Å^2)
			$Yb_{9.19(3)}Ca_{4.81}AlSb_{11}$			
*M*1 [b]	32*g*	0.66(1)/0.34	0.0221(1)	0.1231(1)	0.0056(1)	0.0076(1)
*M*2 [b]	32*g*	0.69(1)/0.31	0.1768(1)	0.2931(1)	0.0791(1)	0.0062(1)
*M*3 [b]	32*g*	0.62(1)/0.38	0.3190(1)	0.0908(1)	0.1580(1)	0.0080(1)
*M*4 [b]	16*e*	0.66(1)/0.34	0.1449(1)	0	1/4	0.0059(2)
Al	8*a*	1	0	1/4	3/8	0.0046(8)
Sb1	32*g*	1	0.0040(1)	0.1126(1)	0.3093(1)	0.0066(1)
Sb2	32*g*	1	0.2752(1)	0.1195(1)	0.2974(1)	0.0066(1)
Sb3	16*f*	1	0.3645(1)	0.6145(1)	1/8	0.0057(1)
Sb4	8*b*	1	0	1/4	1/8	0.0067(2)
			$Yb_{8.42(4)}Ca_{5.58}AlSb_{11}$			
*M*1 [b]	32*g*	0.60(1)/0.40	0.0220(1)	0.1232(1)	0.0051(1)	0.0173(3)
*M*2 [b]	32*g*	0.64(1)/0.36	0.1767(1)	0.2929(1)	0.0790(1)	0.0130(2)
*M*3 [b]	32*g*	0.56(1)/0.44	0.3192(1)	0.0906(1)	0.1580(1)	0.0169(3)
*M*4 [b]	16*e*	0.60(1)/0.40	0.1450(1)	0	1/4	0.0120(4)
Al	8*a*	1	0	1/4	3/8	0.0116(13)
Sb1	32*g*	1	0.0039(1)	0.1124(1)	0.3096(1)	0.0127(2)
Sb2	32*g*	1	0.2755(1)	0.1196(1)	0.2974(1)	0.0124(2)
Sb3	16*f*	1	0.3644(1)	0.6144(1)	1/8	0.0103(2)
Sb4	8*b*	1	0	1/4	1/8	0.0137(3)
			$Yb_{5.12(2)}Ca_{8.98}AlSb_{11}$			
*M*1 [b]	32*g*	0.36(1)/0.64	0.0223(1)	0.1230(1)	0.0056(1)	0.0122(2)
*M*2 [b]	32*g*	0.40(1)/0.60	0.1768(1)	0.2933(1)	0.0791(1)	0.0098(2)
*M*3 [b]	32*g*	0.32(1)/0.68	0.3191(1)	0.0910(1)	0.1580(1)	0.0131(2)
*M*4 [b]	16*e*	0.36(1)/0.64	0.1451(1)	0	1/4	0.0090(3)
Al	8*a*	1	0	1/4	3/8	0.0067(6)
Sb1	32*g*	1	0.0038(1)	0.1131(1)	0.3092(1)	0.0092(1)
Sb2	32*g*	1	0.2752(1)	0.1197(1)	0.2974(1)	0.0092(1)
Sb3	16*f*	1	0.3644(1)	0.6144(1)	1/8	0.0077(1)
Sb4	8*b*	1	0	1/4	1/8	0.0102(2)
			$Yb_{3.43(2)}Ca_{10.57}AlSb_{11}$			
*M*1 [b]	32*g*	0.24(1)/0.76	0.0224(1)	0.1230(1)	0.0061(1)	0.0124(2)
*M*2 [b]	32*g*	0.27(1)/0.73	0.1768(1)	0.2933(1)	0.0792(1)	0.0101(2)
*M*3 [b]	32*g*	0.22(1)/0.78	0.3192(1)	0.0911(1)	0.1580(1)	0.0138(2)
*M*4 [b]	16*e*	0.24(1)/0.76	0.1452(1)	0	1/4	0.0090(3)
Al	8*a*	1	0	1/4	3/8	0.0067(6)
Sb1	32*g*	1	0.0038(1)	0.1132(1)	0.3091(1)	0.0097(1)
Sb2	32*g*	1	0.2751(1)	0.1197(1)	0.2973(1)	0.0095(1)
Sb3	16*f*	1	0.3644(1)	0.6144(1)	0.1250	0.0080(1)
Sb4	8*b*	1	0	1/4	1/8	0.0107(2)

[a] Ueq is defined as one third of the trace of the orthogonalized Uij tensor; [b] M is refined as statistical mixture of Yb and Ca.

Table S2. Selected bond distances (Å) for the Yb14-xCaxAlSb11 (4.81(3) ≤ x ≤ 10.57(2)) series.

Atomic Pair	Distance			
	$Yb_{9.19(3)}Ca_{4.81}AlSb_{11}$	$Yb_{8.42(4)}Ca_{5.58}AlSb_{11}$	$Yb_{5.12(2)}Ca_{8.98}AlSb_{11}$	$Yb_{3.43(2)}Ca_{10.57}AlSb_{11}$
*M*1 [a]-Sb1	3.143(1)	3.154(1)	3.154(1)	3.157(1)
	3.711(1)	3.721(1)	3.724(1)	3.728(1)
*M*1 [a]-Sb2	3.144(1)	3.155(1)	3.156(1)	3.158(1)
	3.264(1)	3.275(1)	3.274(1)	3.276(1)
*M*1 [a]-Sb3	3.252(1)	3.270(1)	3.264(1)	3.264(1)
*M*1 [a]-Sb4	3.403(1)	3.418(1)	3.418(1)	3.419(1)
*M*2 [a]-Sb1	3.207(1)	3.216(1)	3.227(1)	3.231(1)
	3.236(1)	3.243(1)	3.255(1)	3.259(1)
*M*2 [a]-Sb2	3.185(1)	3.197(1)	3.198(1)	3.201(1)
	3.202(1)	3.210(1)	3.218(1)	3.221(1)
*M*2 [a]-Sb3	3.204(1)	3.214(1)	3.221(1)	3.223(1)
*M*2 [a]-Sb4	3.182(1)	3.193(1)	3.197(1)	3.199(1)
*M*3 [a]-Sb1	3.170(1)	3.177(1)	3.180(1)	3.182(1)
	3.455(1)	3.462(1)	3.475(1)	3.479(1)
*M*3 [a]-Sb2	3.214(1)	3.219(1)	3.232(1)	3.236(1)
	3.228(1)	3.237(1)	3.249(1)	3.253(1)
	3.694(1)	3.708(1)	3.716(1)	3.721(1)
*M*3 [a]-Sb3	3.153(1)	3.166(1)	3.165(1)	3.168(1)
*M*4 [a]-Sb1 (x2)	3.267(1)	3.280(1)	3.288(1)	3.292(1)
*M*4 [a]-Sb2 (x2)	3.113(1)	3.127(1)	3.125(1)	3.126(1)
*M*4 [a]-Sb3 (x2)	3.365(1)	3.371(1)	3.383(1)	3.387(1)
Al-Sb1	2.714(1)	2.713(1)	2.712(1)	2.174(1)
Sb3-Sb4	3.190(1)	3.190(1)	3.191(1)	3.193(1)

[a] M is refined as statistical mixture of Yb and Ca.
